# 333. How the COVID-19 Pandemic Accelerated Development of our Complex Outpatient Oral Antimicrobial Therapy (COpAT) Program

**DOI:** 10.1093/ofid/ofad500.404

**Published:** 2023-11-27

**Authors:** Joy J Juskowich, Jesse M Thompson, Arif R Sarwari

**Affiliations:** West Virginia University, Morgantown, West Virginia; West Virginia University, Morgantown, West Virginia; West Virginia University, Morgantown, West Virginia

## Abstract

**Background:**

At our university medical center, we have well established programs for outpatient parenteral antimicrobial therapy (OPAT) and inpatient multidisciplinary antimicrobial infusions for patients with substance use disorder. Complex outpatient oral antimicrobial therapy (COpAT) uses oral (PO) in place of intravenous (IV) antimicrobials. Despite evidence supporting early IV to PO antimicrobial transition for serious infections, COpAT Programs are not widely incorporated in clinical practice. COVID-19 pandemic’s impact on inpatient bed scarcity triggered COpAT’s accelerated implementation at our institution. A Nov 2020 CEO email served as the catalyst and by Aug 2022 an Infectious Diseases (ID) Pharmacist was hired. By Oct 2022, an ID Physician at 0.5 FTE was assigned COpAT Medical Director. We hypothesized IV to PO antimicrobial transition increased as our COpAT Program developed.

**Methods:**

Retrospective cohort study on 380 COpAT patients discharged Jan 2021 to Apr 2023 categorized in 2 groups: *Early Discharge*: Short course IV antimicrobials < 2 weeks followed by COpAT (n= 115); *Traditional*: Longer course IV antimicrobials ≥ 2 weeks followed by COpAT (n= 265). Demographic, clinical, and outcomes data were compared.

**Results:**

Proportion of patients in Early Discharge group increased significantly (19 to 47%, p< 0.001) (Image 1). Traditional group had higher rates of injection drug use and bacteremia. While gram positive organisms predominated in both groups, Traditional group had a higher rate of monomicrobial infection (Image 2). Early Discharge group had a greater proportion of oral antimicrobial days (86 vs. 36%, p< 0.05) and shorter length of stay (8 vs. 24 days, p< 0.05) but higher overall and COpAT-related 30-day readmission rates (15 vs. 6 and 7 vs. 2%, p< 0.05). Both groups avoided inpatient hospital days and had high clinical cure rates (Image 3). COpAT Program ROI was 10.9.

**Figure:**
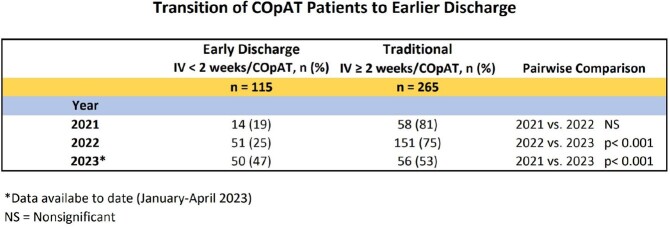

**Figure:**
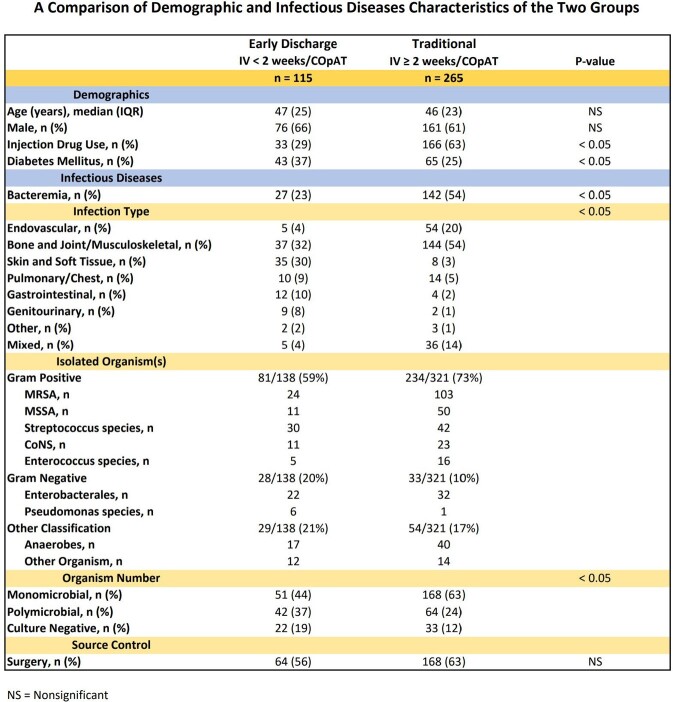

**Figure:**
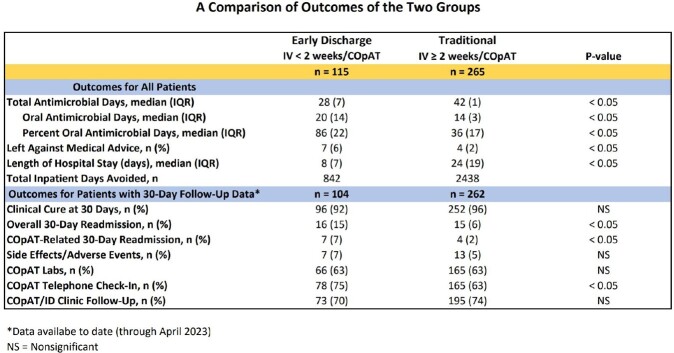

**Conclusion:**

COVID-19 pandemic accelerated development and maturation of our COpAT Program supported by 0.5 FTE ID Physician and 1.0 FTE ID Pharmacist. While readmission rates in both groups were low, possible association between shorter length of stay and increased readmission rates needs further evaluation. COpAT Program investment pays for itself in less than 2 months, mainly through inpatient days avoided.

**Disclosures:**

**All Authors**: No reported disclosures

